# Development of an *in vitro* macrophage screening system on the immunomodulating effects of feed components

**DOI:** 10.1186/s40104-020-00497-4

**Published:** 2020-09-01

**Authors:** S. E. Sivinski, L. K. Mamedova, R. A. Rusk, C. C. Elrod, T. H. Swartz, J. M. McGill, B. J. Bradford

**Affiliations:** 1 Department of Animal Sciences and Industry, Kansas State University, Manhattan, 66506 USA; 2 Department of Diagnostic Medicine/Pathobiology, Kansas State University, Manhattan, 66506 USA; 3 Natural Biologics, Newfield, NY 14867 USA; 4 2265K Anthony Hall, 474 S. Shaw Lane, East Lansing, MI 48824 USA

**Keywords:** Inflammation, *In vitro* screening system, NFκB

## Abstract

**Background:**

While feed components capable of modulating the immune system are highly sought after and marketed, often little evidence is available to support functional immune response claims. Thus, a high-throughput *in vitro* cell screening system was developed to test these compounds for innate immune signaling effects, using *Saccharomyces cerevisiae* and its cell wall components in addition to lauric acid and its esters as models in two separate experiments. This screening system utilized RAW 264.7 murine macrophages to assess live *S. cerevisiae* cells and *S. cerevisiae*-derived cell wall components β-glucan, mannan, and zymosan (a crude cell wall preparation containing both β-glucan and mannan). *D*-mannose was also evaluated as the monomer of mannan. We also examined the effect of a saturated fatty acid (C12:0, lauric acid) and its esters (methyl laurate and glycerol monolaurate) on innate immune cell activation and cellular metabolism. RAW cells were transfected with a vector that drives expression of alkaline phosphatase upon promoter activation of nuclear factor κ-light-chain-enhancer of activated B cells (NFκB), a major inflammatory/immune transcription factor. RAW cells were incubated with 0.01, 0.1 or 1 mg/mL of yeast compounds alone or RAW cells were challenged with LPS and then incubated with yeast compounds. In a separate experiment, RAW cells were incubated with 0, 0.5, 2.5, 12.5, 62.5, and 312.5 μmol/L of lauric acid, methyl laurate, or glycerol monolaurate alone, or RAW cells were challenged with LPS and then incubated with fatty acid treatments.

**Results:**

Treatment with zymosan or β-glucan alone induced NFκB activation in a dose-dependent manner, whereas treatment with *D*-mannose, mannan, or live *S. cerevisiae* cells did not. Post-treatment with mannan after an LPS challenge decreased NFκB activation, suggesting that this treatment may ameliorate LPS-induced inflammation. Slight increases in NFκB activation were found when fatty acid treatments were applied in the absence of LPS, yet substantial reductions in NFκB activation were seen when treatments were applied following an LPS challenge.

**Conclusions:**

Overall, this cell screening system using RAW macrophages was effective, high-throughput, and sensitive to feed components combined with LPS challenges, indicating modulation of innate immune signaling *in vitro*.

**Graphical abstract:**

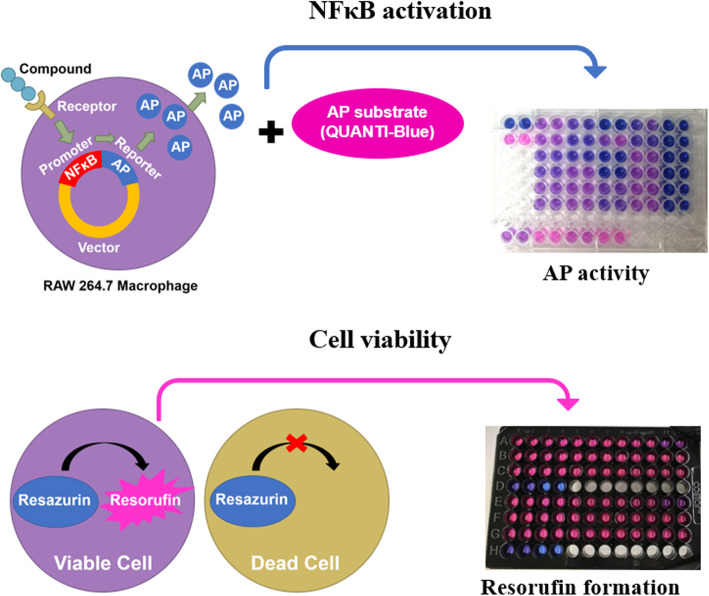

## Background

Compounds with immune-modulating capabilities are of increasing interest [1], but *in vivo *data are expensive and time-consuming to obtain, and thus evidence for feed component-mediated immune effects is often lacking. Therefore, development of an innate immunity *in vitro* cell screening tool would be useful for determining which compounds to investigate *in vivo*. To develop this screening system, *Saccharomyces cerevisiae* and its cell wall components in addition to lauric acid and its esters were used as models in two separate experiments. *Saccharomyces cerevisiae* is a yeast species commonly used in baking and brewing, and is also popularly supplemented in many mammalian diets. The *S. cerevisiae* cell wall, which is 10% to 25% of cell mass, is mostly composed of β-glucans and mannoproteins consisting of approximately 5% protein and 25% to 45% mannan [2–4]. Zymosan, a crude *S. cerevisiae* cell wall extract, contains approximately 50% glucans, 20% mannan, and 15% proteins [5], and is a commonly used immune stimulant. β-Glucans of the *S. cerevisiae* cell wall consist of β-1,3-linked glucose backbones with β-1,6-linked glucose branches [2] while cell wall mannan, which can include up to 200 mannose residues, consists of α-1,6-linked mannose backbones with α-1,2- and α-1,3-linked branches with up to three mannose residues [6, 7]. These components, along with *S. cerevisiae* cells themselves, impact several aspects of immunity both *in vitro* and *in vivo* [8]. Similarly, lauric acid (LA), a medium chain fatty acid (C12:0), and its esters (glycerol monolaurate and methyl laurate) have bactericidal [9–14], anti-inflammatory [15], and antioxidant properties [16]. Thus, these feed components serve as models of interest for development of this* in vitro* screening system.

One central way these feed compounds can influence immunity is through activation of nuclear factor kappa-light-chain-enhancer of activated B cells (NFκB). Nuclear factor κB is an inducible central regulator of inflammatory responses involved in most innate immune receptor signaling pathways, triggering expression of pro-inflammatory cytokines such as tumor necrosis factor α (TNFα). The canonical pathway of NFκB activation involves signaling via pattern recognition receptors, including Toll-like receptors (TLR) and C-type lectin-like receptors (e.g., mannose receptor; MR) found on mammalian cells [17, 18]. After receiving these signals, the inhibitor of kappa B (IκB) becomes phosphorylated, thus leading to its degradation, and allowing nuclear translocation of NFκB [19]. Reviews on NFκB signaling in inflammation and immunity have been published previously [19–21].

The widely-used RAW 264.7 murine macrophage cell line [22, 23] is useful for assessing broad impacts on inflammatory/immune signaling, because the cells are armed with a wide variety of pathogen-associated molecular pattern receptors. We used RAW cells stably transfected with a vector that drives expression of an alkaline phosphatase (AP) reporter gene upon activation of the response elements for NFκB and activator protein 1.

The primary objective of the current study was to determine immune-modulating properties of *S. cerevisiae* components as well as lauric acid and its esters in RAW 264.7 murine macrophages. The secondary objective was to develop a high-throughput innate immune *in vitro* cell screening tool capable of identifying potential immune-modulating compounds. Our hypothesis was that *S. cerevisiae* cells and their major cell wall component, β-glucan, would elicit the strongest immune modulation. Similarly, we hypothesized that lauric acid and its esters would attenuate immune activation.

## Materials and methods

### Cell culture protocol

RAW-Blue™ cells were derived from RAW 264.7 macrophages (Cat. No. raw-sp; Invivogen, Manassas, VA, USA). These cells were stably transfected with a vector that drives expression of an AP reporter gene upon activation of the response elements for NFκB and activator protein 1. First, thawed cells were transferred to RAW cell initial growth media, which consisted of Minimum Essential Media with 4.5 g/L glucose (MEM; Cat No. 11095080; Thermo Fisher Scientific, Waltham, MA, USA), 10% heat-inactivated fetal bovine serum (FBS), 100 μg/mL Normocin (Cat. No. ant-nr-1; Invivogen), and 2 mmol/L *L*-glutamine (Cat. No. 25030081; Thermo Fisher Scientific). Cells were centrifuged at 300×*g* for 5 min, then the supernatant was discarded and cells were resuspended in 1 mL initial growth media. Cells were then seeded at 2.5 × 10^6^ cells in initial growth media per 75 cm^2^ flask. Cells were maintained at 37 °C. Upon reaching approximately 80% confluency, RAW cells were passaged by gently scraping cells from the flask surface, centrifuging at 300×*g* for approximately 7 min, discarding the supernatant, resuspending and centrifuging as before, and finally resuspending in RAW cell maintenance media and seeding them in 75 cm^2^ flasks. RAW cell maintenance media consisted of MEM with 4.5 g/L glucose, 10% heat-inactivated FBS, 2 mmol/L *L*-glutamine, 1% antibiotic-antimycotic (Cat. No. 15240062; Thermo Fisher Scientific), 100 μg/mL Normocin, and 200 μg/mL Zeocin (Cat. No. ant-zn-1; Invivogen). Cells were passaged again upon achieving approximately 80% confluency. Using a hemocytometer (Cat. No. 02-671-54; Hausser Scientific, Horsham, PA, USA), RAW cells were counted under a microscope at 10× magnification. Cells were plated at 1 × 10^5^ cells per well in clear 96-well plates (Cat. No. 353072; Corning, Corning, NY, USA) for AP activity or black-sided, clear-bottom 96-well plates (Cat. No. 3603; Corning) for measuring resorufin formation.

In general, reagents used for cell culture and for experiments were the highest purity compounds available from suppliers. However, LPS content was not specifically evaluated in all reagents, and some limited contamination cannot be excluded.

### Yeast experiment

Compounds were added in a dose-titration (log scale) progression. Treatments were 0.01, 0.1, or 1 mg/mL of β-glucan (Cat. No. 34-621-025MG; Fisher Scientific, Waltham, MA, USA), *D*-mannose (Cat. No. M6020-25G; Sigma-Aldrich, St. Louis, MO, USA), mannan (Cat. No. M7504-250MG; Sigma-Aldrich), live yeast cells (type II, Cat. No. YSC2-500G; Sigma-Aldrich), or zymosan (cat. no. Z4250-250MG; Sigma-Aldrich). Treatment concentrations were chosen to represent a broad spectrum of potential doses for the RAW cell system within a range of what has been tested *in vitro* [24–26]. β-Glucan, mannan, and zymosan were derived from *S. cerevisiae*. The live *S. cerevisiae* cell treatment was dried by the manufacturer to produce 90% viable, active cells. Compounds were dissolved in phosphate-buffered saline (PBS), except for β-glucan, which was suspended in 0.1 mmol/L sodium bicarbonate. This solution did not alter NFκB activation or cellular metabolism compared to PBS diluent (data not shown). To assess the capacity of yeast compounds to aid RAW cells in recovering from an LPS challenge, RAW cells were incubated with 0.1 μg/mL lipopolysaccharide (LPS; Cat. No. L4391; Sigma-Aldrich) for 6 h. Because LPS is known to stimulate NFκB activation, all 180 μL of media was then replaced to remove the confounding AP already secreted into cell supernatants. Yeast treatments (*n* = 6 replicates per treatment) were then added directly to the fresh media on top of RAW cells and incubated for 4 h. Experiments assessing the direct effects of yeast treatments alone followed the same protocol previously mentioned but without LPS stimulation during the first 6 h. Unstimulated RAW cells and 0.1 μg/mL LPS-stimulated cells served as controls.

### Lauric acid experiment

Treatments were 0, 0.5, 2.5 12.5, 62.5, and 312.5 μmol/L lauric acid (LA; Cat. No. PHR1580; Sigma-Aldrich), glycerol monolaurate (GML; Natural Biologics, Newfield, NY, USA), or methyl laurate (ML; Natural Biologics). Compounds were dissolved in 0.06% DMSO (ATCC, Manassas, VA, USA). Cells were challenged with LPS (Cat. No. L9023; Sigma-Aldrich) for 6 h, media was then removed, and lipid treatments were applied for an additional 4 h (*n* = 6 replicates per treatment). Experiments assessing the direct effects of fatty acid treatments alone followed the same protocol previously mentioned but without LPS stimulation during the first 6 h. Unstimulated RAW cells and 0.1 μg/mL LPS-stimulated cells served as controls. To access potential DMSO effects, we included additional control groups, where cells were only treated with 0.06% DMSO or DMSO with 0.1 μg/mL LPS.

### Cellular metabolism

Cellular metabolism was assessed by resazurin metabolism [27]. In black-walled, clear bottom 96-well plates, 20 μL of a 0.15 mg/mL solution of resazurin sodium salt (Cat. No. R7017-5G; Sigma-Aldrich) was added directly to RAW cells in 200 μL of media following treatments. These plates were then incubated for another 4 h. In a preliminary experiment, we assessed the coefficient of variation at 2 and 4 h, and the 4 h time period was chosen as it minimized inter-assay CV (data not shown). Cellular metabolism was assessed by measuring fluorescent resorufin formation in relative fluorescent units (RFU) after 4 h of incubation with excitation at 560 nm and 590 nm emission using a fluorometric plate reader (Synergy HTX; BioTek Instruments Inc., Winooski, VT, USA) and Gen5 software (BioTek Instruments Inc.).

### NFκB activation

50 μL of cell media from each well was transferred to another 96-well plate containing 150 μL of AP substrate solution per well (QUANTI-Blue; Cat. No. rep-qb1; Invivogen) and incubated for 2 h. In a preliminary experiment, AP activity was assessed every 15 min starting 30 min until 6 h after the addition of AP substrate solution. The 2 h time point was determined to be suitable as all enzymatic AP curves were increasing but none had reached a plateau (data not shown). To assess NFκB/activator protein 1 activation, AP activity was quantified by determining optical density (OD) at 620 nm with a colorimetric plate reader (Synergy HTX; BioTek Instruments Inc.) and calculations were performed using Gen5 software (BioTek Instruments Inc.).

### Process optimization

Additional developments in the RAW cell culturing process were required. This included testing multiple sources of FBS because certain FBS sources had background AP activity (data not shown), despite the fact that all FBS sources were heat-inactivated. To test this background variation, 5 μL of FBS was added to 45 μL of MEM to replicate 10% of FBS in RAW maintenance media, as would be present in 50 μL of RAW cell supernatant when transferred to QUANTI-Blue AP substrate media. This 50 μL mixture was then added to 150 μL of AP substrate media and incubated for 2 h. Absorbance was then measured as previously described. In addition to differences across suppliers, FBS sources from the same company but from different lot numbers had different AP activity. Observed AP activity of media containing GIBCO exosome-free FBS (Cat No. A2720803; ThermoFisher Scientific) did not differ from that of PBS or MEM alone (*P* > 0.05) and was thus used for RAW cell experiments.

### Statistical analysis

Data were analyzed using PROC GLIMMIX (SAS 9.4, SAS Institute Inc.). Fixed effects of compound, LPS, and their interactions were evaluated, with plate as a random effect. If a variable required transformation to meet the assumption of normality, back-transformed means and standard errors are reported according to Jørgensen and Pedersen [28]. Studentized residuals greater than the absolute value of 4 were excluded from analysis. Linear and quadratic contrasts were conducted to evaluate dose-dependent effects. When a factor was found to be significant in the overall model (*P* <  0.05), treatment means were separated using Tukey’s HSD test.

## Results

### Yeast experiment

**Fig. 1:**
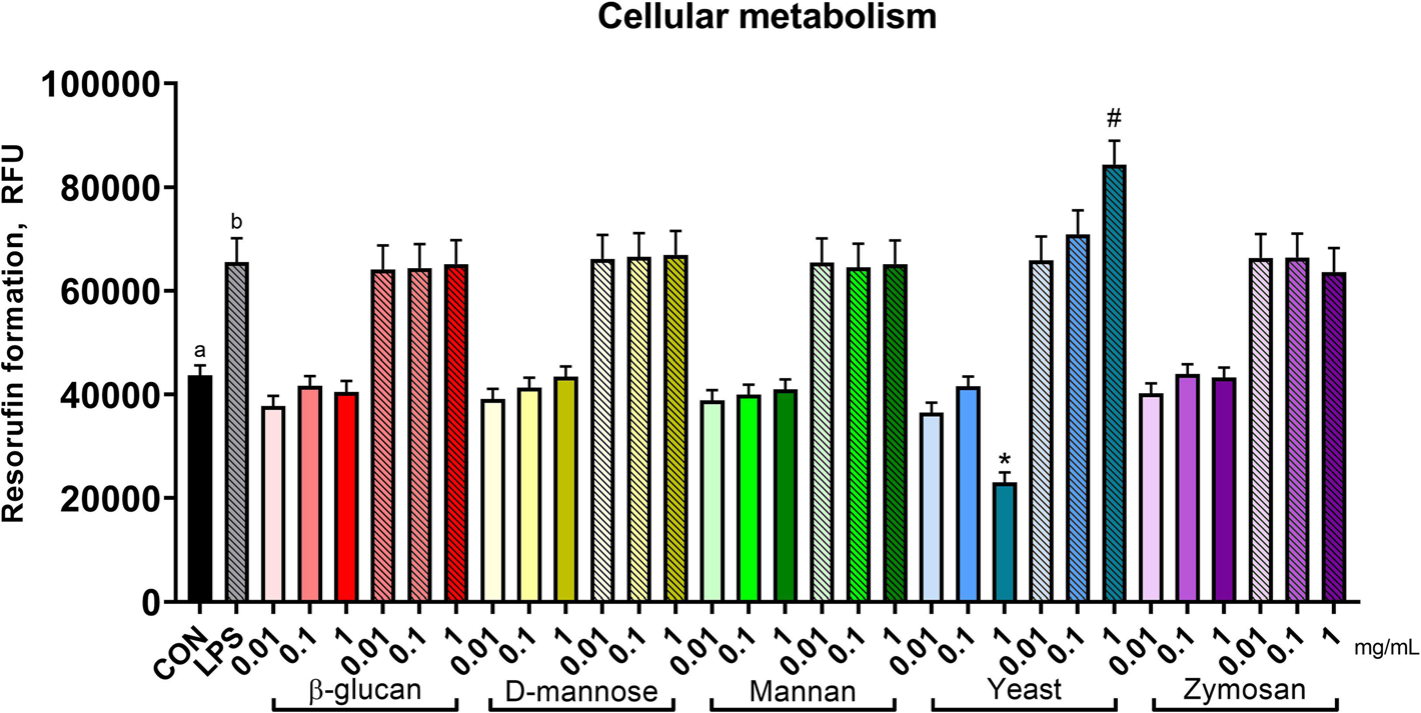
Metabolic activity of murine macrophages after treatment with components of *Saccharomyces cerevisiae*. Metabolic activity was assessed by metabolism of resazurin to the fluorescent product resorufin. Untreated RAW cells represent the negative control, and RAW cells stimulated with 0.1 μg/mL LPS served as the positive control. Increasing doses (0.01, 0.1, or 1 mg/mL) are signified by increasing color intensity within treatment compound. Solid bars indicate control or cells treated with yeast components, whereas patterned bars indicate cells stimulated with LPS or both LPS and yeast components. Values are reported as LSM ± SE. * indicates means (yeast components alone) differ significantly from CON, # indicates means (yeast components following LPS) differ significantly from LPS (*P* < 0.05, Tukey’s HSD). Different letters indicate means differ (*P* < 0.05, Tukey’s HSD). Overall, yeast treatment interacted with LPS stimulation (interaction, *P* < 0.01) on cellular metabolism of resazurin

The effect of yeast and yeast components on cellular metabolic activity depended on LPS (Fig. 1; compound × LPS, *P* <  0.01). Where live yeast cells alone reduced cellular metabolism at the 1 mg/mL concentration (1 mg/mL live *S. cerevisiae* vs. CON, *P* <  0.01), the same concentration of live yeast cells increased metabolism during an LPS challenge (1 mg/mL live *S. cerevisiae* LPS vs. CON-LPS, *P* <  0.01; quadratic effect yeast × LPS, *P* <  0.01; Table 1). No other effects of yeast or yeast components were found on cellular metabolism; LPS increased cellular metabolism as compared to untreated cells (*P* <  0.01).

**Table 1:** Linear and quadratic contrasts and interaction contrasts of yeast and yeast components

	Compound contrasts										Compound × LPS contrasts									
	β-glucan		*D*-mannose		Mannan		Yeast		Zymosan		β-glucan		*D*-mannose		Mannan		Yeast		Zymosan	
	L^a^	Q^b^	L	Q	L	Q	L	Q	L	Q	L	Q	L	Q	L	Q	L	Q	L	Q
Cellular metabolic activity	0.55	0.18	0.60	0.21	0.42	0.20	0.69	0.72	0.77	0.88	0.66	0.62	0.79	0.18	0.67	0.32	< 0.01	< 0.01	0.48	0.21
NFκB activation	< 0.01	< 0.01	< 0.01	< 0.01	0.86	0.66	0.88	< 0.01	< 0.01	< 0.01	< 0.01	< 0.01	< 0.01	< 0.01	< 0.01	< 0.01	< 0.01	< 0.01	< 0.01	< 0.01

^a^*L* linear contrast

^b^*Q* quadratic contrast

**Fig. 2:**
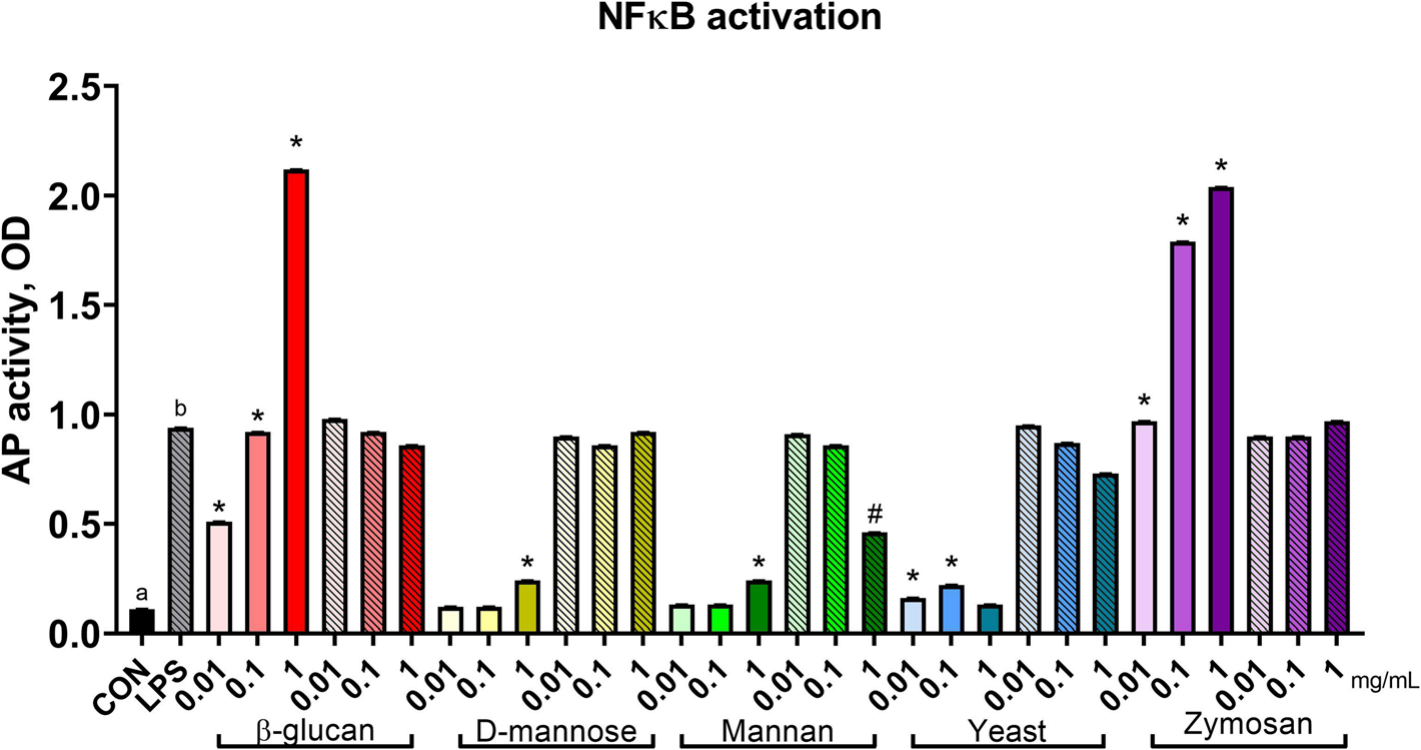
Nuclear factor κB activation of murine macrophages treated with components of *Saccharomyces cerevisiae*. Activity of NFκB was assessed with a reporter plasmid driving expression of alkaline phosphatase (AP). Untreated RAW cells represent the negative control, and RAW cells stimulated with 0.1 μg/mL LPS served as the positive control. Increasing doses (0.01, 0.1, or 1 mg/mL) are signified by increasing color intensity within treatment compound. Solid bars indicate control or cells treated with yeast components, whereas patterned bars indicate cells stimulated with LPS or both LPS and yeast components. Values were back transformed and reported as LSM ± SE. * indicates means (yeast components alone) differ significantly from CON, # indicates means (yeast components following LPS) differ significantly from LPS (*P* < 0.05, Tukey’s HSD). Different letters indicate means differ (*P* < 0.05, Tukey’s HSD). Overall, yeast treatment interacted with LPS stimulation (*P* < 0.01) on NFκB activation

For NFκB activation, yeast and yeast components interacted with LPS (Fig. 2; compound × LPS, *P* <  0.01). Quadratic relationships (Table 1) were identified where every yeast and yeast component (all *P* <  0.01) altered NFκB activation in the absence of an LPS challenge, yet neutral effects or suppression of NFκB activity were observed when treatments were applied following an LPS challenge. In particular, β-glucan and zymosan at every concentration, as well as *D*-mannose and mannan (1 mg/mL) and live yeast cells (0.01 and 0.1 mg/mL) increased NFκB activation as compared to untreated controls. When RAW cells were stimulated with LPS, 1 mg/mL mannan reduced NFκB activation when compared to LPS controls (*P* <  0.01).

### Lauric acid experiment

**Fig. 3:**
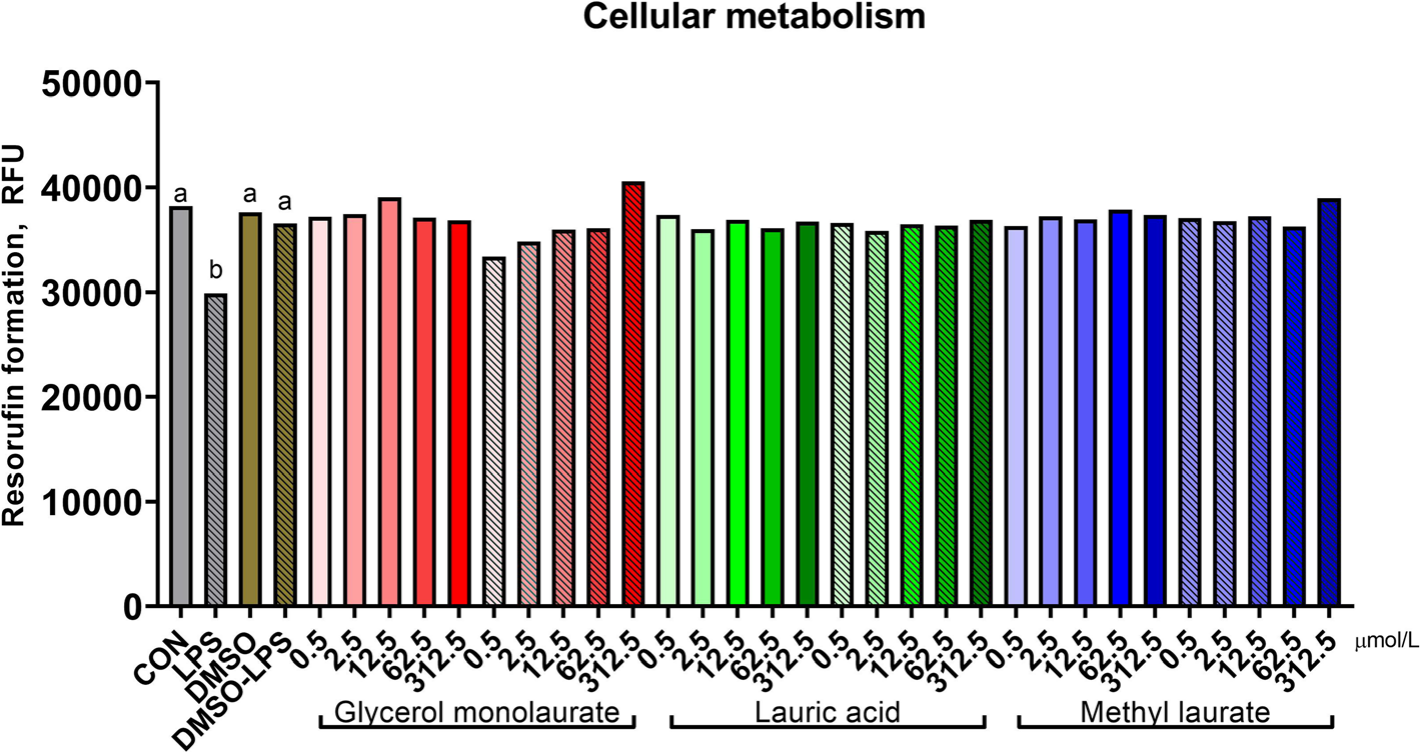
Metabolic activity of murine macrophages after treatment with lauric acid or its esters. Metabolic activity was assessed by metabolism of resazurin to the fluorescent product resorufin. Untreated RAW cells represent the negative control, and RAW cells stimulated with 0.1 μg/mL LPS served as the positive control. Increasing doses (0, 0.5, 2.5, 12.5, 62.5, and 312.5 μM) are signified by increasing color intensity within treatment compound. Solid bars indicate control or cells treated with glycerol monolaurate, lauric acid, or methyl laurate, whereas patterned bars indicate cells stimulated with LPS or both LPS and fatty acids. Values are reported as LSM ± SE. * indicates means (fatty acids alone) differ significantly from DMSO, # indicates means (fatty acids following LPS) differ significantly from DMSO-LPS. Different letters indicate means differ (*P* < 0.05, Tukey’s HSD). Overall, fatty acid treatment interacted with LPS stimulation (interaction, *P* < 0.01) on cellular metabolism of resazurin

The effect of lauric acid and its esters on metabolic activity depended on LPS (Fig. 3; compound × LPS, *P* <  0.01). Quadratic relationships were identified for GML (*P* <  0.01), as treatment had little impact on metabolic activity in the absence of an LPS challenge, yet dose-dependently increased it when GML was applied following an LPS challenge (Table 2). No other fatty acid treatment altered cellular metabolic activity. LPS reduced metabolic activity; however, DMSO attenuated this response (*P* <  0.01).

**Table 2:** Linear and quadratic contrasts and interaction contrasts of lauric acid and its esters

	Compound contrasts						Compound × LPS contrasts					
	Glycerol monolaurate		Lauric acid		Methyl laurate		Glycerol monolaurate		Lauric acid		Methyl laurate	
	L^a^	Q^b^	L	Q	L	Q	L	Q	L	Q	L	Q
Cellular metabolic activity	< 0.01	0.01	0.49	0.23	0.13	0.21	< 0.01	< 0.01	0.31	0.87	0.42	0.67
NFκB activation	< 0.01	< 0.01	< 0.01	< 0.01	< 0.01	< 0.01	< 0.01	< 0.01	< 0.01	< 0.01	< 0.01	< 0.01

^a^*L* linear contrast

^b^*Q* quadratic contrast

**Fig. 4:**
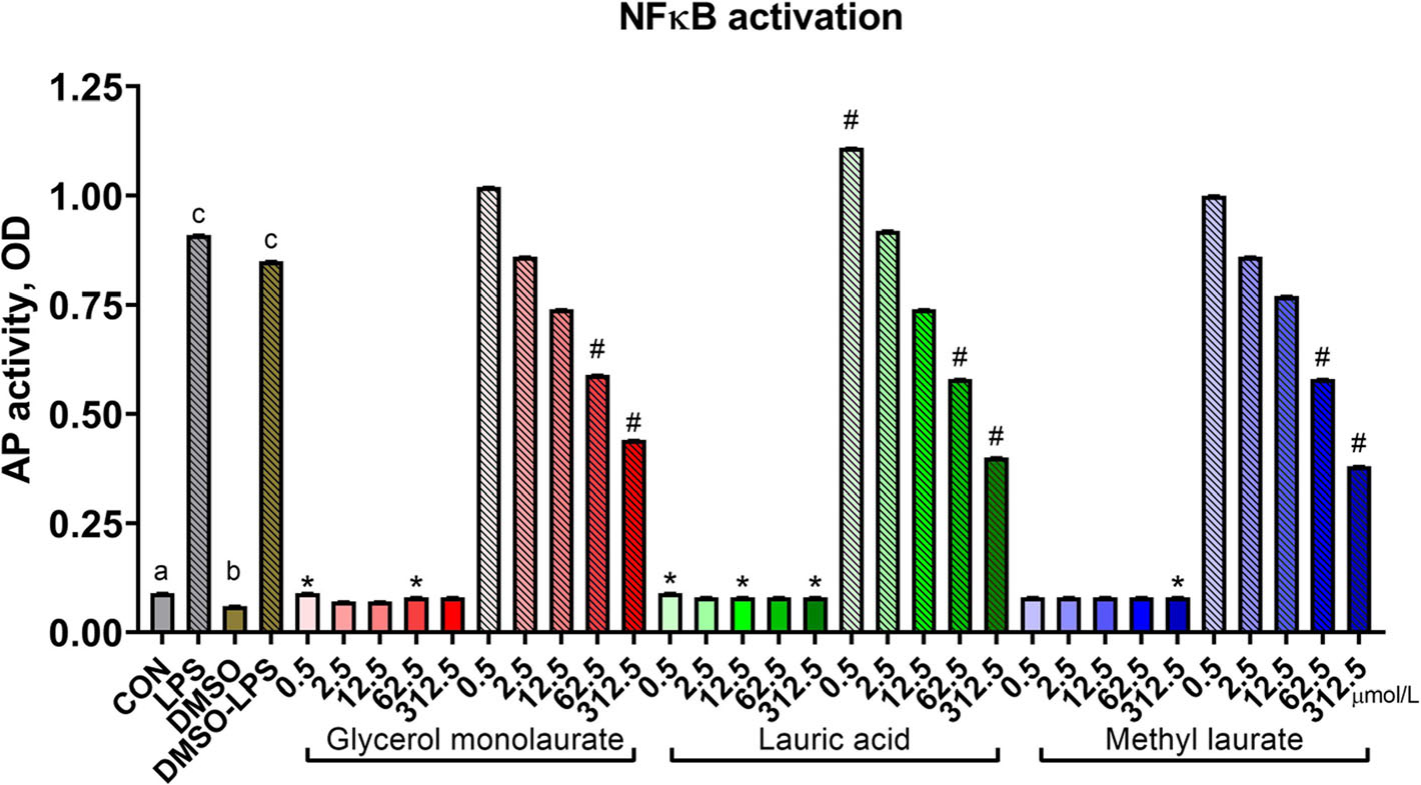
Nuclear factor κB activation of murine macrophages treated with lauric acid or its esters. Activity of NFκB was assessed with a reporter plasmid driving expression of alkaline phosphatase (AP). Untreated RAW cells represent the negative control, and RAW cells stimulated with 0.1 μg/mL LPS served as the positive control. Increasing doses (0, 0.5, 2.5, 12.5, 62.5, and 312.5 μmol/L) are signified by increasing color intensity within treatment compound. Solid bars indicate control or cells treated with glycerol monolaurate, lauric acid, or methyl laurate, whereas patterned bars indicate cells stimulated with LPS or both LPS and fatty acids. Values are reported as LSM ± SE. * indicates means (fatty acids alone) differ significantly from DMSO, # indicates means (fatty acids following LPS) differ significantly from DMSO-LPS. Different letters indicate means differ (*P* < 0.05, Tukey’s HSD). Overall, fatty acid treatment interacted with LPS stimulation (interaction, *P* < 0.01) on NFκB activation

For NFκB activation, lauric acid and its esters interacted with LPS (Fig. 4; compound × LPS, *P* <  0.01). Quadratic relationships were identified where lauric acid, methyl laurate, and glycerol monolaurate (all *P* < 0.01) slightly increased NFκB activation in the absence of an LPS challenge, with a far more dramatic dose-dependent reduction in NFκB activation when treatments were applied following an LPS challenge. Specifically, 62.5 and 312.5 μmol/L concentrations of GML, ML, and LA reduced NFκB activation as compared to the DMSO-LPS group (all *P* < 0.01). Similar to its impact on metabolic activity, DMSO reduced NFκB activation when compared to untreated cells (*P* < 0.01), although this effect was not found in LPS-challenged cells (*P* = 0.96).

## Discussion

### Effect of yeast and yeast components on RAW cellular metabolism and activation

Our objective was to develop an *in vitro* system to test the effects of feed components on immune cell activation. First, we examined the effects of yeast and yeast components alone as well as following an LPS challenge on macrophage cellular metabolism and NFκB activation. Although β-glucan, *D*-mannose, mannan, and zymosan did not affect cellular metabolism, resazurin metabolism was decreased by treatment with live yeast cells at 1 mg/mL vs. control. Roy et al. [24] reported lactate dehydrogenase leakage and thus decreased cell viability of murine wound macrophages with exposure to *S. cerevisiae*-derived β-glucan at 100 μg/mL, but not at 20 μg/mL. In contrast, we did not observe any decreases in cellular metabolism with β-glucan across different doses used in our study.

NFκB activation was affected by yeast compounds in the absence of an LPS challenge. Zymosan is known to induce NFκB activation in macrophages [29–31]; thus, our RAW cell results paralleled previously published results. Walachowski et al. [25] determined that zymosan stimulated the greatest NFκB activation in RAW 264.7 murine macrophages, with similar responses to another crude *S. cerevisiae* cell wall extract that contained 15% β-glucan, 33% proteins, and 19% mannans, among other compounds. In contrast, the *S. cerevisiae* cell wall extracts containing 65% or 75% β-glucan, which also consisted of 3.7% and < 1% mannan, respectively, weakly induced NFκB activation. These results contrast observations with our purified, particulate β-glucan source, as well as those reported by McCann et al. [32]. Walachowski et al. [25] also found that TLR2 and TLR4 crosstalk with dectin-1 stimulated greater NFκB activity with β-glucan stimulation. Similarly, Roy et al. [24] reported that *S. cerevisiae*-derived β-glucan activated NFκB via TLR2 and dectin-1 in macrophages isolated from murine wounds. Soluble β-glucan from *S. cerevisiae* can activate NFκB in human [33] and murine [34] macrophage cell lines, though results have not been consistent [25].

Recognition of β-glucan and zymosan involves dectin-1 and TLR2 [35–37]. Though zymosan contains mannan, a polymer of mannose, the mannose receptor was not predominant in zymosan recognition by macrophages [38]. RAW macrophages also required expression of myeloid differentiation primary response gene 88 (MyD88) to activate NFκB in response to curdlan, a linear particulate β-1,3-glucan derived from bacteria [34, 39]. Though *S. cerevisiae* β-glucans are β-1,3-1,6-glucan, a 99% pure linear β-1,3-glucan was derived from *S. cerevisiae* and similarly stimulated NFκB activation and expression of pro-inflammatory cytokines [40]. This linear β-1,3-glucan interacted with TLR2 and complement receptor 3 (CR3). In contrast, blocking CR3 did not affect zymosan recognition by macrophages, suggesting that CR3 recognition depends on structure. However, zymosan required dectin-1, TLR2, and MyD88 to induce TNFα production in murine peritoneal and RAW 264.7 macrophages [35]. Interestingly, RAW 264.7 murine macrophages were shown to express only low levels of dectin-1; greater expression of the receptor led to greater zymosan-induced TNFα production [35]. However, Walachowski et al. [25] reported that dectin-1 expression could be enhanced by incubation with β-glucans, which further increased TNFα production by this macrophage cell line.

Treatment with *D*-mannose, mannan, and live yeast cells had marginal effects on NFκB activation. Interestingly, live *S. cerevisiae* cells induced greater macrophage binding to the yeast cells and greater TNFα production than live *C. albicans* [35], which could indicate greater NFκB activation as well [29]. Mannan from *S. cerevisiae* induced TNFα production by human monocytes, and required CD14 and TLR4, but not TLR2 [26]; these results suggest that mannan may have activated NFκB, the inflammatory transcription factor for TNFα [19, 20, 41]. The mannose receptor (MR) is expressed on macrophages, including RAW 264.7 macrophages [42], and can strongly bind mannose via the manosyl / fucosyl calcium-dependent recognition pattern [43–45]. As mannan has been considered a ligand for this pathway, *S. cerevisiae* mannan could also bind to MR on macrophages to elicit effects [43, 45, 46]. *D*-mannose is also taken up by alveolar macrophages [47] and is recognized by the macrophage MR [46].

### Effect of yeast compound treatment after an LPS challenge

Cellular metabolism was affected by yeast compounds following an LPS challenge. Post-treatment at 1 mg/mL with live yeast cells resulted in the greatest metabolic activity. Interestingly, while cellular metabolism increased with yeast cell dose, no effect was detected for NFκB activation. Thus, changes in cellular metabolism or viability were most likely not related to NFκB activation. We speculated that the high apparent metabolism of RAW cells exposed to live yeast may have been due to metabolic activity of the yeast itself, and we indeed found that yeast placed in media in the absence of RAW cells showed substantial metabolism of resazurin (data not shown).

Yeast compounds affected NFκB activation following an LPS challenge. Mannan post-treatment at 1 mg/mL decreased NFκB activity compared to the LPS control, suggesting that mannan could be used as a remedial treatment to LPS-induced inflammation. While literature is lacking on the topic, simultaneous incubation with LPS and mannan from *S. cerevisiae* did not act synergistically to induce TNFα production in human monocytes, though each compound did elicit this effect independently [26]. In further support of an inflammatory/immuno-regulatory role of mannan from *S. cerevisiae*, Che et al. [48] observed lesser TNFα production and greater IL-10 production by porcine alveolar macrophages incubated with mannan oligosaccharide. In contrast, post-treatment with β-glucan, *D*-mannose, live yeast cells, and zymosan did not result in different NFκB activation compared to the positive control. This result suggests that these yeast compounds are unable to ameliorate LPS-induced inflammatory signaling, but also do not further potentiate LPS-induced NFκB activation post LPS-challenge.

### Effect of lauric acid esters on cellular metabolism and NFκB activation

We examined the effect of lauric acid and its esters on macrophage cellular metabolism and NFκB activation. Resazurin metabolism of RAW macrophages was only marginally affected by fatty acid treatments, with a quadratic effect found with glycerol monolaurate. Past studies have found no effect of lauric acid on macrophage cell viability at lower concentrations (less than 20 μmol/L for 24 h incubation) [49], although another study with a 48 h incubation period found cytotoxic effects at greater concentrations (400 μmol/L) [50]. Judging from these studies, lauric acid likely impacts cell viability but only at greater concentrations with longer incubation times than those used in the present study.

Similar to cellular metabolism, lauric acid and its esters had minimal impacts on NFκB activation in the absence of an LPS challenge, although at some concentrations statistically significant increases were found. Past studies discovered that lauric acid increased NFκB activation through TLR2 dimerization with TLR1 or 6, as well as through TLR4 activation [51–54]. Indeed, lauric acid induced dimerization of TLR4 and MD-2 in lipid rafts, which is the initial step in TLR4 signal transduction [55]. However, a recent study demonstrated that another saturated fatty acid, palmitic acid, did not directly activate TLR4 nor did it induce dimerization of TLR4 and MD-2; rather its proinflammatory effects are mediated through shifts in lipidomic profiles due to reprogramming of macrophage metabolism [56]. Similar considerations may be necessary in interpreting signaling responses to lauric acid.

### Effect of lauric acid and its esters after an LPS challenge

Lauric acid and its esters did not affect cellular metabolism during an LPS challenge. Intriguingly, LPS reduced cellular metabolism, but DMSO attenuated this response. Because LPS is a pro-inflammatory factor that can have cytotoxic effects, it seems likely that this was abolished by the anti-inflammatory effect of DMSO [57]. When comparing the negative control to the LPS positive control, it is interesting that in the yeast experiment LPS increased cellular metabolism, but in the fatty acid experiment LPS reduced cellular metabolism. Endotoxin sources were different between experiments, suggesting that LPS-induced cellular metabolic responses may be variable due to *Escherichia coli* strain differences (LPS from *E. coli* O55:B5 used in the lauric acid experiment vs. LPS from *E. coli* O111:B4 used in the yeast experiment).

While fatty acid treatment marginally increased NFκB activation in the absence of an LPS challenge, a sizeable dose-dependent reduction occurred following an LPS challenge. A similar effect was found in microglia post-LPS challenge with lauric acid concentrations of 100 to 200 μmol/L [16], paralleling our study conducted in RAW cells. This biphasic response is likely due to activation of GPR40, a fatty acid receptor [58]. When GPR40 is activated, nitric oxide synthase and reactive oxygen species (ROS) production is partially reduced [16], however, it is still unknown whether this is a direct effect of lauric acid or some other agonist such as a lauric acid metabolite. A reduction in ROS would reduce inflammatory responses and promote resolution, potentially explaining the basis for lauric acid inhibition of NFκB activity. Moreover, lauric acid treatment reduced pro-inflammatory cytokine concentrations, TNFα, IL-6, and IL-1β, in cell culture medium from LPS-challenged microglia [16], corroborating our findings that lauric acid reduced NFκB activation in RAW cells. Collectively, these data suggest that lauric acid treatment along with its esters could be used to promote resolution following an inflammatory insult.

### Limitations

This RAW macrophage screening system is based on one simple measurement *in vitro*, and thus has limitations on what information can be collected. *In vitro* analysis is limited to certain aspects of inflammatory/immune signaling which cannot replicate the many signaling interactions found *in vivo*. Most obviously, only one immune cell type was used. *Saccharomyces cerevisiae* and its components also impact other innate immune cells, including dendritic cells and neutrophils [31, 59, 60]. These cell types also contain NFκB and pattern recognition receptors that respond to *S. cerevisiae* components; thus, stimulation with *S. cerevisiae* components generally results in similar outcomes as macrophages. However, the lack of parenchymal cells as well as adaptive immune cells such as B and T lymphocytes may result in missing functional effects of compounds. In this simple cell screening system, potential changes in structure of feed components through the digestive process are lacking, and the bioavailability of some of these components also remains unknown; thus, concentrations of components used in this screening system may not reflect what would be available to immune cells* in vivo*.

While acknowledging these limitations, our results nonetheless demonstrate the promise of using this screening approach to enable researchers to focus on biomolecules with the greatest potential to influence immune responses. We evaluated yeast components in part because of the strong literature around these compounds both *in vitro* and *in vivo*. Consistent with our findings, β-glucans result in immune modulation *in vivo* across a wide range of concentrations and species [61–63]. Had this system been used for an initial assessment of components of yeast cells to determine which component to focus on, it likely would have have pointed in the most fruitful direction for further *in vivo* testing.

## Conclusions

The *in vitro* cell screening system using RAW 264.7 murine macrophages was an accurate, quick, sensitive model for determining effects of *Saccharomyces cerevisiae* and fatty acid components on innate immunity. Assessing cellular metabolic activity also helped to determine if changes in NFκB activation due to treatments were related to changes in amount of viable, metabolically-active RAW macrophages. When RAW cells were treated with various yeast compounds alone, zymosan and β-glucan stimulated the greatest NFκB activation, consistent with many previous reports in various cell types. The use of yeast treatments following an LPS challenge, in particular mannan at 1 mg/mL, was able to attenuate LPS-induced NFκB activation. Lauric acid had minimal impacts on cellular metabolism, regardless of LPS challenge. However, substantial reductions in NFκB activation were found when lauric acid was applied following an LPS challenge. In general, the most dramatic results appeared when compounds were tested as a “recovery” agent after endotoxin challenge as compared to their effects alone. Overall, this 2-step *in vitro* cell screening system was able to detect differences between treatments on macrophages particularly following an endotoxin challenge, suggesting that this may be a fruitful screening strategy.

## Data Availability

The datasets used and analysed during the current study are available from the corresponding author on reasonable request.
